# Effectiveness of germicidal ultraviolet light to inactivate coronaviruses on personal protective equipment to reduce nosocomial transmission

**DOI:** 10.1017/ice.2021.249

**Published:** 2022-07

**Authors:** Carolina Camargo, Andréanne Lupien, Fiona McIntosh, Dick Menzies, Marcel A. Behr, Selena M. Sagan

**Affiliations:** 1 Department of Microbiology & Immunology, McGill University, Montreal, Québec, Canada; 2 Department of Medicine, Research Institute of the McGill University Health Centre, McGill University, Montreal, Québec, Canada; 3 McGill International TB Centre, McGill University, Montreal, Québec, Canada; 4 Department of Biochemistry, McGill University, Montreal, Québec, Canada

**Keywords:** Germicidal Ultraviolet light (GUV), Coronavirus (CoV), SARS-CoV-2, COVID-19, N95 respirator, Personal Protection Equipment (PPE)

## Abstract

**Objective:**

To circumvent the need for rationing personal protective equipment (PPE), we explored whether germicidal ultraviolet light (GUV) could be used to inactivate human coronaviruses on PPE, enabling safe reuse.

**Design:**

We performed a laboratory study to assess the ability of 2 commercially available portable GUV devices to inactivate 2 common cold coronaviruses (HCoV-229E and HCoV-OC43) and severe acute respiratory syndrome coronavirus virus 2 (SARS-CoV-2), which causes coronavirus disease 2019 (COVID-19), on the surface of whole N95 respirators and coupons cut from those respirators. We experimentally contaminated N95 respirators with coronavirus cultures and then assessed viral inactivation after GUV exposure by plaque assay, the median tissue culture infectious dose (TCID_50_) assay, and quantitative reverse-transcriptase polymerase chain reaction (RT-PCR).

**Results:**

We found that GUV could efficiently inactivate coronaviruses on the surface of N95 masks, with an average reduction in viral titers of 5-log for HCoV-229E, 3-log for HCoV-OC43, and 5-log for SARS-CoV-2. In addition, the GUV susceptibility of HCoV-229E was similar on coupons and whole N95 respirators.

**Conclusions:**

We demonstrate that diverse human coronaviruses, including SARS-CoV-2, are susceptible to GUV inactivation, and 2 scalable portable GUV devices were effective in inactivating coronaviruses on N95 respirators. Thus, GUV treatment with commercially scalable devices may be an effective method to decontaminate PPE, allowing their safe reuse.

The susceptibility of microorganisms to germicidal ultraviolet light (GUV) has been well established experimentally, although the susceptibility of severe acute respiratory coronavirus virus 2 (SARS-CoV-2), which causes coronavirus disease 2019 (COVID-19), was first estimated by extrapolation from other coronaviruses.^1–3^ The doped-quartz–based GUV lamps in use today filter out wavelengths below 240 nm. Direct exposure to GUV radiation has important safety hazards, so its use in hospitals, clinics, and laboratories, has been limited to indirect exposure, such as irradiation of air leaving tuberculosis patient rooms.

Recent pandemics of respiratory viral illnesses, particularly SARS-CoV-1 (2003), H1N1 (2009), and SARS-CoV-2 (2019) have demonstrated that shortages of personal protective equipment (PPE) can occur due to sudden massive increases in demand, far exceeding stored supplies and manufacturing capacity.^4,5^ Already, the COVID-19 pandemic has created severe shortages of PPE in hospitals around the world, which has led to rationing. In general, however, healthcare workers have had high rates of infection due to ineffective protection (eg, breaches in availability) or complete lack of PPE (eg, in which infection was not suspected or occurred outside the healthcare setting).^6^ The prediction of shortages during a pandemic resulted in experimental work, led by the US Centers for Disease Control (CDC) and National Institute for Occupational Safety and Health (NIOSH), to determine whether N95 respirators and other PPE could be safely disinfected. Our results confirm 4 findings: (1) GUV can disinfect N95 respirators,^7^ although the rate of success depends upon the characteristics of the mask. (2) Dirt and saliva can accumulate on respirators and reduce efficacy. (3) The optimal exposure for disinfection for almost all N95 models under almost all conditions is 1 joules per cm^2^ (J/cm^2^), achieving >3-log_10_ reductions of H1N1 influenza virus, generally thought to be less susceptible to GUV than coronaviruses.^8^ And (4) repeated GUV exposure (ie, 10 or 20 times) does not significantly reduce respirator filtration ability, strap elasticity, resistance to air flow, or physical integrity.^7,9^

Substantial experimental work has demonstrated the feasibility and efficacy of GUV to disinfect N95 respirators; however, this was accomplished using custom-made devices in experimental labs. Hence, no production model has been tested for its ability to adequately and safely disinfect N95 respirators or other PPE. Additionally, the GUV susceptibility of SARS-CoV-2 is likely, based on genetic similarity to related coronaviruses, but this requires confirmation. Herein, we tested several human coronaviruses (HCoV), including HCoV-229E (α-coronavirus), HCoV-OC43 (β-coronavirus), and SARS-CoV-2 (β-coronavirus) for susceptibility to GUV. We used local manufacturers of GUV devices (Sanuvox Technologies and LifeAire Systems LLC), who provided 2 portable devices for decontamination. We experimentally contaminated N95 respirators with coronaviruses and evaluated viral inactivation after GUV treatment in cell culture. Both devices were effective at inactivating coronaviruses, and both support the use of GUV as a practical solution to address PPE shortages.

## Material and methods

### Cells and viruses

Huh-7 cells were obtained from C. M. Rice and were cultured as described previously.^10^ HCT-8 and VeroE6 cells (ATCC) were grown in Dulbecco’s modified Eagle medium (DMEM) with 10% heat-inactivated fetal bovine serum (FBS), L-glutamine, and nonessential amino acids. HCoV-229E (ATCC) was propagated and titered in Huh-7 cells at 33°C under a 5% CO_2_ atmosphere, based on reported optimal growth conditions.^8,11^ For propagation, infections were performed for 2 hours in OPTI-MEM (Life Technologies) with rocking every 15 minutes. The inoculum was then removed and replaced with infection media (complete DMEM + 2% FBS). When cytopathic effects (CPE) were apparent in ∼80% of cells (∼5 days after infection), viral supernatants and cell-associated virus were recovered using 3 freeze–thaw cycles. Cellular debris was pelleted by centrifugation, and viral stocks were recovered and stored at −80°C. HCoV-OC43 (ATCC) was propagated in HCT-8 and titered in Vero-E6 cells as described above.

SARS-CoV-2 (isolate RIM-1, Genbank accession no. MW599736) was isolated from a quantitative reverse-transcriptase polymerase chain reaction (PCR)–positive patient sputum sample at the RI-MUHC using tosyl phenylalanyl chloromethyl ketone (TPCK)–treated VeroE6 cells as previously described.^12^ Viral stocks prepared in VeroE6 cells at 37^o^C under 5% CO_2_. Supernatants were collected 3 days after infection, were concentrated 5–10-fold using an Amicon Ultra-15 centrifugal filter unit (100 KDa cutoff), and stored at −80°C. All cell lines were routinely screened for mycoplasma contamination using the MycoAlert mycoplasma detection kit (Lonza).

### Plaque assays

HCoV-229E was titered by plaque assay using 10-fold serial dilutions in Huh-7 cells. Infections were carried out as for viral propagation, with the exception that 2% methylcellulose was included in the media to restrict viral diffusion. At 5 days after infection, cells were washed with phosphate-buffered saline (PBS) and fixed with formal saline for 1 hour at RT. Cells were washed with ddH_2_O and stained with 0.1% crystal violet for 30 minutes. Excess of stain was removed, and viral titers (in plaque-forming units, PFU) were estimated using equation 1:
(1)

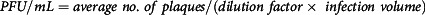




### *TCID*_*50*_ *assays*

For HCoV-OC43 and SARS-CoV-2, sample titers were measured using the median tissue culture infectious dose (TCID_50_) assay using 10-fold serial dilutions in Vero-E6 cells. Cells were infected for 1 hour, after which the inoculum was removed and replaced with infection media. After 3 or 5 days for SARS-CoV-2 and HCoV-OC43, respectively, cells were fixed and stained as described above. Viral titers were calculated using the the Reed-Muench method that estimates TCID_50_/mL.^13^

### Quantitative RT-PCR analysis

For total RNA isolation, infection was monitored for CPE and cells were harvested at day 4 after infection. Briefly, cells were washed with PBS, lysed in TriZol, and total RNA was isolated according to the manufacturer’s instructions. Two sets of primers (targeting the RNA-dependent RNA polymerase (RdRp) and Nucleocapsid (N) genes) were used for gene-specific reverse transcription and amplification, except for SARS-CoV-2, which was analyzed using RdRp only (Supplementary Table 1 online). Complementary DNA (cDNA) was synthesized with M-MuLV RT (NEB) using 500 ng of total RNA, according to the manufacturer’s instructions. Quantitative real-time PCR amplification was carried out using SYBR Green technology with the iTaq Universal SYBR Green PCR master mix according to the manufacturer’s instructions. Genome copies were calculated using a standard curve, and fold differences in gene expression were calculated using the 2^–ΔΔCt^ method.^14^

### HCoV inactivation assays

Using procedures established by NIOSH, 2 × 2-cm coupons were cut from sterile N95 respirators in the biological safety cabinet (BSC) and affixed with a single staple. Coupons were placed in a 6-well tissue culture dish and 20 × 3 µL droplets of HCoV-229E stock (4.8 × 10^8^ PFU/mL), HCoV-OC43 (1.67 × 10^4^ TCID_50_/mL), or SARS-CoV-2 (1 × 10^7^ TCID_50_/mL) were placed on each coupon. Coupons were dried in the BSC for 0.5–1 hour and were either untreated (no UV) or treated with GUV using the small (Sanuvox Technologies) or large (LifeAire Systems LLC) devices. The small device is a cardboard box [38 cm × 30.5 cm × 24 cm (15 inches × 12 inches × 9.5 inches); 1.3 kg (3 pounds)] with 2 U-shaped bulbs (above and below) and capacity for 1 respirator suspended by its straps. The exposure time can be manipulated as needed. The large device is a polished aluminum box [109 cm × 71 cm × 61 cm (43 inches × 28 inches × 24 inches)] with a capacity of ∼20 N95 respirators. Both devices are designed to deliver 0.5 to 1.0 J/cm^2^.

For the small UV device, coupons in a 6-well plates were placed in the center of the unit and exposed to UV for 2.5 minutes. For the large device, coupons were treated for 2.5 minutes, flipped with sterile tweezers, and treated for another 2.5 minutes (UV delivery is top down in this device). UV exposure was confirmed using UVC 1,000 dosimeters, and according to the indicator, coupons were exposed to 0.5 J/cm^2^. Coupons were then rehydrated in 1.5 mL OPTI-MEM for 1 hour at RT and the media was recovered using a cell strainer (40 µm) and centrifugation at 4,000 rpm for 2 minutes. For HCoV-229E, recovered virus was titered by plaque assay; for HCoV-OC43 and SARS-CoV-2, recovered virus was titered by TCID_50_ assay. Log reductions were calculated using equation 2:
(2)

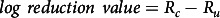

where *R*_*c*_ is mean viable virus recovered from control coupons (log PFU/mL or log TCID_50_) and *R*_*u*_ is viable recovery from UV-exposed coupons (log PFU/mL or log TCID_50_).

For whole-mask testing, intact N95 respirators (folded particulate respiratory, SAS Safety Corp) were spotted with HCoV-229E (as described above) in 4 zones: (1) nose, (2) right cheek, (3) left cheek, and (4) chin. Contaminated respirators were subjected to GUV treatment, then coupons were cut and processed as described above.

### Data analysis

Statistical analyses were performed using GraphPad Prism version 7 software (GraphPad, San Diego, CA). No a priori statistical power calculation was conducted. Sample size was based on similar previous studies.^4,15,16^ All data are representative of 3 independent experiments with 3 independent replicates per experiment (n = 9). All data points represent 3 independent measurements per replicate, and error bars represent standard deviation (SD). Statistical significance was determined using an unpaired *t* test.

## Results

### UV inactivation of HCoV-229E on N95 respirator coupons

**Fig. 1.**
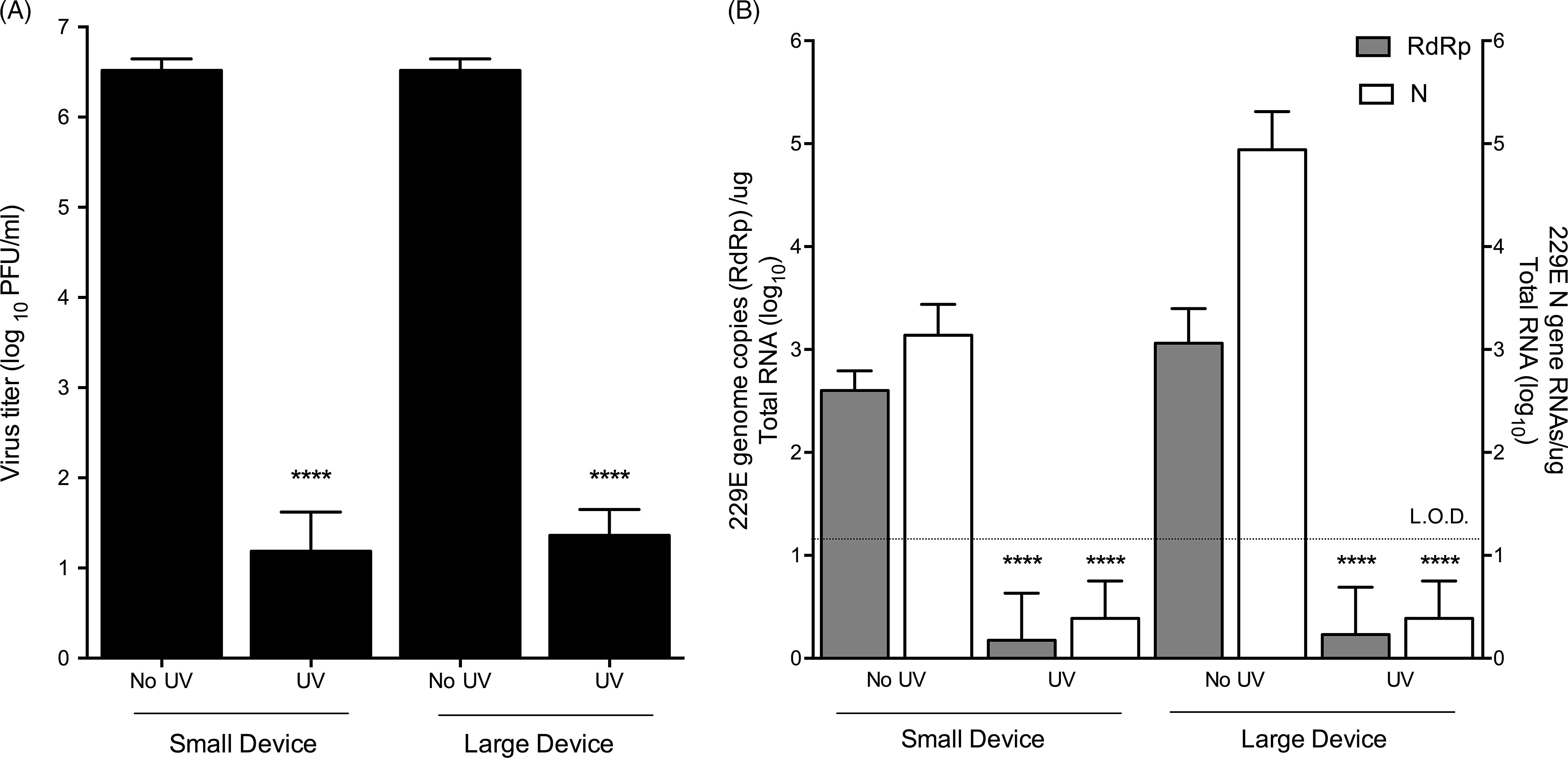
HCoV-229E can be effectively inactivated by GUV exposure. (A) Plaque assay after UV treatment of HCoV-229E-contaminated coupons in small or large UV devices. (B) qRT-PCR analyses of HCoV-229E after UV treatment using RdRp or N gene-specific primers. All data are representative of 3 independent experiments with three technical replicates per experiment (n = 9) and error bars represent SD. Statistical significance was determined using an unpaired *t* test.

To test the efficacy of UV inactivation of HCoV-229E on N95 respirators, coupons were inoculated with HCoV-229E. After drying, coupons were UV treated, and any remaining virus was recovered and quantified via plaque assay and qRT-PCR analyses (Fig. 1). In untreated (no UV) samples, HCoV-229E remained viable through the drying process and was efficiently recovered from the coupons, generating an average titer of 3.35 × 10^6^ PFU/mL (Fig. 1A). UV treatment was effective at reducing HCoV-229E titers by ∼5-log in both UV devices (Fig. 1A). These results were validated by qRT-PCR analysis of viral growth in cell culture (Fig. 1B). Specifically, qRT-PCR analysis using the viral RdRp and N genes demonstrated that while we were readily able to detect viral RNAs in control (no UV) samples, after UV-treatment viral RNAs were below the limit of detection (Fig. 1B). These results suggest that UV treatment provides an effective means of inactivating the common cold alphacoronavirus, HCoV-229E.

### UV inactivation of HCoV-OC43 on N95 respirator coupons

**Fig. 2.**
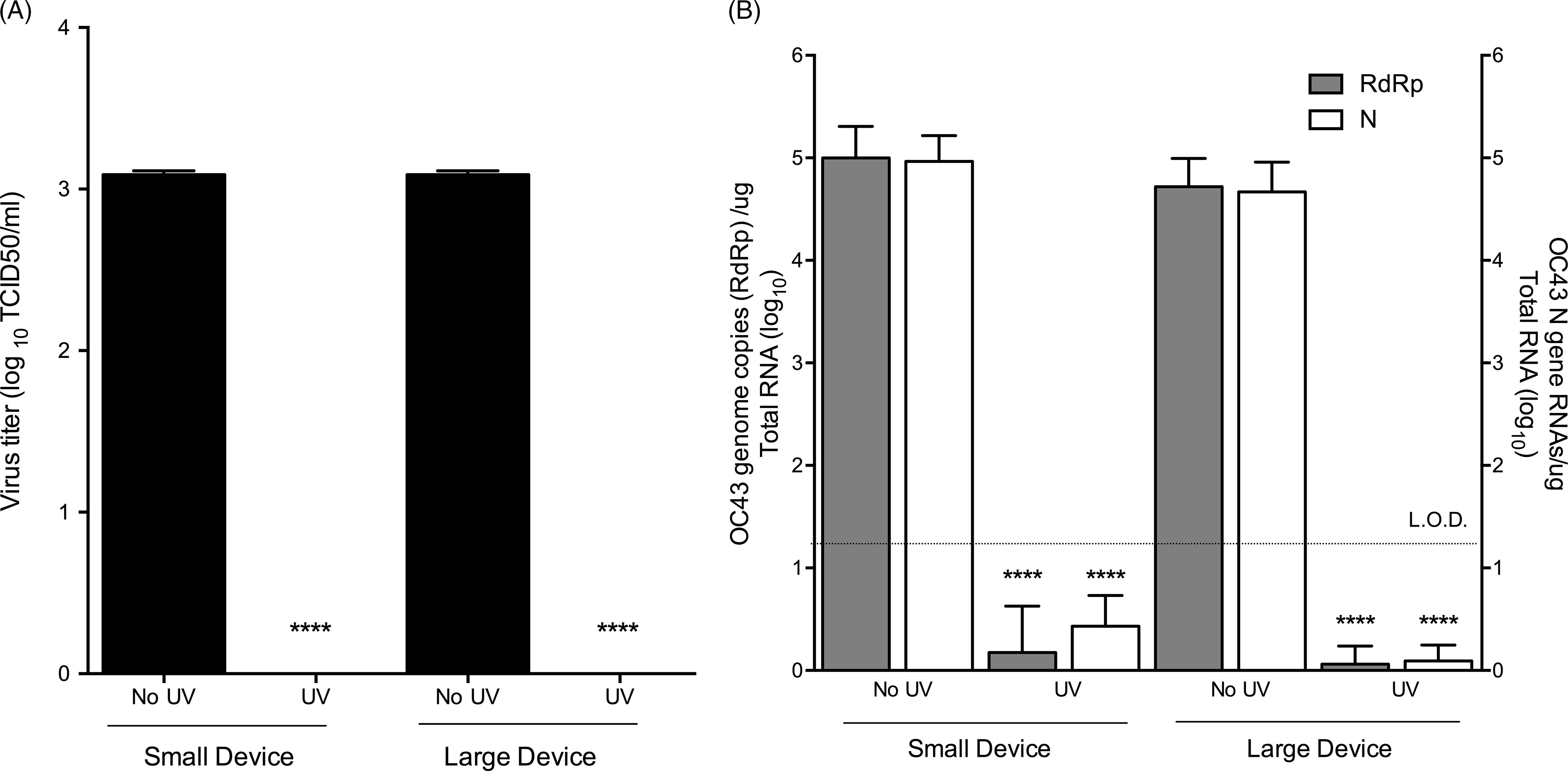
HCoV-OC43 can be effectively inactivated by GUV exposure. (A) TCID_50_ assay after UV treatment of HCoV-OC43–contaminated coupons. (B) qRT-PCR analyses of HCoV-OC43 after UV treatment using RdRp or N gene-specific primers. Data are representative of 3 independent experiments with three technical replicates per experiment (n = 9). Error bars represent SD. Statistical significance was determined using an unpaired *t* test.

To test the efficacy of UV inactivation of HCoV-OC43 on N95 respirators, we followed a similar procedure as described for HCoV-229E, except that TCID_50_ assays were used for quantification of infectious viral titers (Fig. 2). Similarly, HCoV-OC43 was efficiently recovered from coupons in the untreated (no UV) samples, with an average titer of 1.33 × 10^3^ TCID_50_/mL (Fig. 2A). UV treatment reduced HCoV-OC43 titers by ∼3-log for both UV devices (Fig. 2A), which was further corroborated by qRT-PCR analysis (Fig. 2B). As observed with HCoV-229E, in all UV-treated samples, HCoV-OC43 viral RNAs were below the limit of detection via qRT-PCR (Fig. 2B). Thus, similarly as for HCoV-229E, UV treatment provides an effective means of inactivating the common cold β-coronavirus, HCoV-OC43.

### UV inactivation of SARS-CoV-2 on N95 coupons

**Fig. 3.**
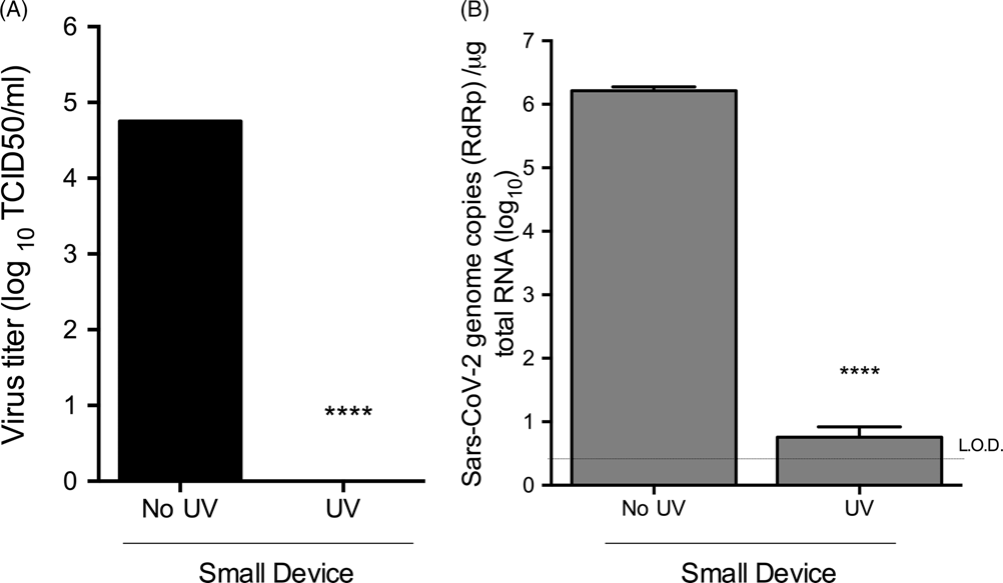
SARS-CoV-2 can be inactivated by GUV exposure. (A) TCID_50_ assay with after UV treatment of SARS-CoV-2–contaminated coupons in small or large UV devices. (B) qRT-PCR analyses of SARS-CoV-2 after UV treatment using RdRp gene-specific primers. Data are representative of 3 independent experiments with 3 technical replicates per experiment (n = 9). Error bars represent SD. Statistical significance was determined using an unpaired *t* test.

To test the efficacy of UV inactivation of SARS-CoV-2 on N95 respirators, we followed a similar procedure as described above using TCID_50_ assays for quantification of infectious viral titers. As SARS-CoV-2 experiments were carried out in Biosafety Level 3 (BSL3) containment, only the small UV device was used (Fig. 3). SARS-CoV-2 remained viable through the drying process and was efficiently recovered generating an average titer of 5.6 × 10^4^ TCID_50_/mL. UV treatment reduced SARS-CoV-2 titers by ∼5-log (Fig. 3A). In all UV-treated samples, SARS-CoV-2 viral RNAs were below the limit of detection (Fig. 3B). Thus, UV treatment provides an effective means of inactivating SARS-CoV-2.

### Effectiveness of UV inactivation on whole respirators

**Fig. 4.**
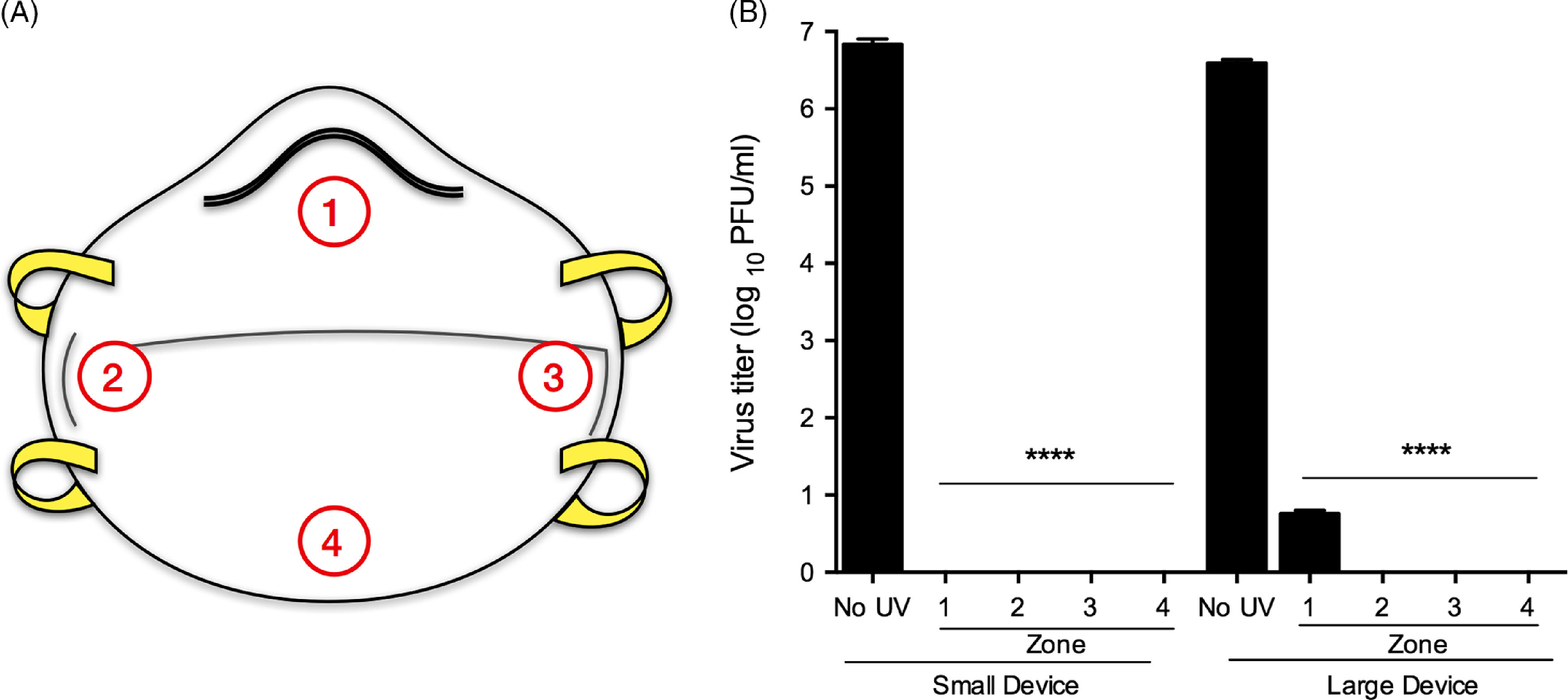
GUV inactivation of HCoV-229E on intact N95 respirators is subject to differential efficacy based on location of inoculation. (A) Graphical representation of the zones on the N95 respirators that were spotted with microdroplets of HCoV-229E. Zones 1–4 represent (1) nose, (2) right cheek, (3) left cheek, and (4) chin. (B) Plaque assay after UV treatment of HCoV-229E-contaminated respirators, separated by zone in the small or large UV devices. Data are representative of 3 independent experiments with 3 technical replicates per experiment (n = 9). Error bars represent SD. Statistical significance was determined using an unpaired *t* test.

The efficacy of GUV inactivation is dependent upon direct exposure to the target surface and can be influenced by surface type as well as creases or folds on N95 respirators. To test whether this might alter UV inactivation efficacy, we contaminated and UV-treated intact (whole) respirators (Fig. 4). We selected HCoV-229E for these studies due to its high titer, and therefore largest dynamic range. HCoV-229E was spotted on 4 different zones of the N95 respirator: (1) nose, (2) right cheek, (3) left cheek, and (4) chin (Fig. 4A). Whole respirators were then subjected to UV treatment in both UV devices, coupons were cut, and viral titers were assessed by plaque assay (Fig. 4B). For both devices, UV treatment resulted in a significant reduction in viral titers, with average log reductions of 6-log and 5-log, for the small and large UV devices, respectively (Fig. 4B). Interestingly, while UV treatment with the small UV device demonstrated no significant differences in UV inactivation by zone, in the large UV device, we observed complete inactivation in zones 2–4, but we recovered viable virus from zone 1 in multiple replicates (nose, Fig. 4B). However, we observed less than 10 PFUs in all cases, suggesting that we nevertheless observed a significant decrease in viral titer in zone 1 (nose), with on average >5-log reductions in viral titers.

## Discussion

The susceptibility of microorganisms to GUV suggests that decontamination protocols can be used to attain greater usage of disposable masks and respirators during epidemics, where there is a surge in demand. Herein, we found that UV treatment was an effective means of inactivating common cold coronaviruses (HCoV-229E and HCoV-OC43) and SARS-CoV-2 on the surface of N95 respirators. Specifically, we observed average log reductions in infectious viral titers of 3-log (HCoV-OC43) and 5-log (HCoV-229E and SARS-CoV-2). However, the more modest 3-log reduction in HCoV-OC43 titers may be related to the low starting titer (1.67 × 10^4^ TCID_50_/mL) of this virus, which generated low titer stocks in our hands. Importantly, we also validated these results by assessment of viral gene expression in coronavirus-infected cells by qRT-PCR using both the RdRp and N genes.^17^ Although the RdRp gene is only present on genome-length viral RNAs, the N gene is present on both genome-length and subgenomic mRNAs. Independent examination of gene expression by these 2 gene products helped to validate our findings and has previously been shown to minimize both false-positive and false-negative results.^18^ Notably, measurements of infectious particles, such as PFU and TCID_50_, show a strong correlation with cycle threshold values obtained by real-time PCR over a wide range of virus titers.^7^ Thus, taken together, our results suggest that coronaviruses are highly susceptible to GUV-mediated inactivation. This finding also agrees with previous reports that suggest that respiratory pathogens, including viruses (eg, influenza) and fungi (eg, *Candida*) are highly susceptible to UV inactivation.^4,19^

In this study, we evaluated a variety of human coronaviruses that are known to cause outbreaks (ie, HCoV-229E, HCoV-OC43, and SARS-CoV-2) to provide a broader depiction of the effects of GUV treatment of PPE with several distinct but related respiratory viruses. Several models have demonstrated differential susceptibility to GUV according to viral structure and nucleic acid content, with single-stranded RNA viruses being the most susceptible and double-stranded DNA viruses the least susceptible.^20^ Under this spectrum and due to the single-stranded, RNA-based nature of the coronavirus genome, human coronaviruses are predicted to be highly susceptible to GUV.^20^ Another consideration is the genomic G+C content, which for human coronaviruses values range between 32% and 43%, considered on the lower end for RNA viruses, and in theory may make them susceptible to GUV inactivation.^21^

Although previous studies have suggested that GUV can be used to disinfect N95 respirators, the rate of success depends upon the characteristics of the mask (ie, deeply folded surfaces may render GUV less effective since it relies on direct exposure).^5,21^ Previous studies suggest that this can be overcome by using longer exposure times or by achieving higher UV exposures (>1 J/cm^2^). To assess this, we tested GUV treatment on whole (intact) N95 respirators. Some locations on the N95 respirator were slightly less susceptible to GUV when treated in the large device, which could be a function of the N95 respirator design itself, to the position of the UV lamps in this device (top down), or a combination of both factors. However, in both GUV devices, even at ∼0.5 J/cm^2^, we were able to significantly impact viral inactivation (independent of zone), with at least 3-log reductions for each coronavirus, which has been previously reported as an effective threshold for pathogen decontamination.^5,21^ Thus, both GUV devices tested herein are portable units that could potentially overcome the limitations of using laboratory-designed units.^22^

Importantly, our study has several limitations, the most significant being that it was performed in vitro using cell-culture–grown coronaviruses, and may overlook elements of real-life nosocomial contamination. First, the viral titers used herein likely exceed what is generated in patient fluids during a clinical contamination event.^23–25^ Specifically, for SARS-CoV-2, pharyngeal shedding is very high during the first week of symptoms, with a peak at 7.11 × 10^8^ RNA copies per throat swab on day 4 after infection.^25^ Recent comparative analyses of molecular diagnostic assays suggest that genome copies exceed PFU counts in the range of 1000:1.^24^ In addition, previous models have predicted contamination levels from aerosol (<5 µm) sources in hospital settings to be the highest at 10^5^ PFU/mL for influenza virus and 10^6^ TCID_50_/mL for SARS-CoV-1.^26^ Thus, the viral titers generated in cell culture herein likely exceed those generated in patient fluids. Second, UV efficacy is dependent upon direct exposure to the target surface and is known to be influenced by the presence of soiling agents (ie, bodily fluids) and was not assessed in this study.^21^ Finally, while we attempted to address direct exposure to the target surface, our findings cannot necessarily be extrapolated to all brands of N95 respirators, all coronaviruses, or all types of GUV devices. However, the agreement in inactivation thresholds between the 2 GUV devices tested suggests that effective inactivation can be achieved with devices with varied design considerations. Although previous studies have indicated that repeated GUV exposure does not significantly reduce mask filtration ability, strap elasticity, resistance to airflow, or physical integrity,^7,9,21^ it is possible that some mask components could begin to degrade over time, which is likely to be model specific. As such, limiting reuse to 5 donnings has been proposed as an adequate safety margin.^27^ Nonetheless, it is of the utmost importance for users to carefully monitor the integrity of reused respirators to ensure proper functioning and user protection.

In conclusion, we have demonstrated that diverse human coronaviruses, including SARS-CoV-2, are susceptible to GUV inactivation. Our results indicate that GUV treatment with commercially scalable devices may be effective to decontaminate N95 respirators from human coronavirus droplets to allow safe reuse of PPE.

## Financial support

This research was supported by funding from the McGill Interdisciplinary Initiative in Infection and Immunity (MI4) Emergency COVID-19 Research Funding (ECRF) Program and the MUHC Foundation. In addition, this research was undertaken, in part, thanks to the Canada Research Chairs program (to S.M.S., M.B., and D.M.). SARS-CoV-2 experiments were conducted in containment level 3 technology platform at RI-MUHC, Montreal, Québec, Canada.

## Supplementary material

For supplementary material accompanying this paper visit https://doi.org/10.1017/ice.2021.249. click here to view supplementary material
