# Supplementary material for: Effectiveness of germicidal ultraviolet light to inactivate coronaviruses on personal protective equipment to reduce nosocomial transmission
Source: Infect Control Hosp Epidemiol. 2021 Jun 21;43(7):886–91. doi: 10.1017/ice.2021.249 (PMC9272544; doi:10.1017/ice.2021.249)
Supplement: Supplementary file 1 [file S0899823X2100249Xsup001.docx]

**Supplementary Information**

**Table S1. qRT-PCR primers used in the study.**

|  | **Target Gene** | **Amplicon Size (nt)** | **Primer sets (5’-3’)** |
| --- | --- | --- | --- |
| **229E** | RdRp | 272 | TTTTTCTGTCGCTGCTTTGA  ATAGCGAGCTGCCACTTGAT |
|  | N | 216 | TCTGCCAAGAGTCTTGCTCG  AGCATAGCAGCTGTTGACGG |
| **OC43** | RdRp | 228 | CATGTTTTGGGCCTCTTGTT  CGGCAACACTAAAACAGCAA |
|  | N | 282 | CCCAAGCAAACTGCTACCTCTCAG  GTAGACTCCGTCAATATCGGTGCC |
| **SARS-CoV-2** | RdRp | 128 | CGCTTCCAAGAAAAGGACGA  GCAACAGCTGGACAATCCTT |
| **Control** | GAPDH | 226 | GAAGGTGAAGGTCGGAGTC  GAAGATGGTGATGGGATTTC |
